# Optical activity levels of metal centers controlling multi-mode emissions in low-dimensional hybrid metal halides for anti-counterfeiting and information encryption†

**DOI:** 10.1039/d4sc05041j

**Published:** 2024-09-16

**Authors:** Qiqiong Ren, Guojun Zhou, Yilin Mao, Nan Zhang, Jian Zhang, Xian-Ming Zhang

**Affiliations:** a Key Laboratory of Magnetic Molecules and Magnetic Information Materials (Ministry of Education), School of Chemistry and Material Science, Shanxi Normal University Taiyuan 030031 P. R. China zhangxm@dns.sxnu.edu.cn; b College of Chemistry & Chemical Engineering, Key Laboratory of Interface Science and Engineering in Advanced Material, Taiyuan University of Technology Taiyuan Shanxi 030024 P. R. China

## Abstract

In-depth insight into the electronic competition principles between inorganic units and organic ligands proves to be extremely challenging for controlling multi-mode emissions in low-dimensional hybrid metal halides (LHMHs). Herein, an efficient blue emission from organic ligand was engineered in (DppyH)_2_MCl_4_ (Dppy = diphenyl-2-pyridylphosphine, M = Zn^2+^, Cd^2+^) due to the reverse type I band alignment constructed by optically inert units with nd^10^ shell electrons. By contrast, the optically active [MnCl_4_]^2−^ with semi-fully filled 3d^5^ shell electrons prompts the band alignment of type II, resulting in the narrowband green emission of Mn^2+^, along with an energy transfer from DppyH^+^ to [MnCl_4_]^2−^. Beyond that, the band alignment of (DppyH)SbCl_4_ is further reversed to type I due to the strong stereochemical activity of 5s^2^ lone-pair electrons, resulting in the triplet-state (^3^P_1_ → ^1^S_0_) self-trapped exciton (STE) emission of [SbCl_4_]^−^. The conclusion is that the electronic configurations of metal centers govern the optical activity levels of inorganic units, which in turn controls the multi-mode emissions by maneuvering the band alignments. This research provides an enlightening perspective on the multi-mode emissions with tunable photoluminescence and resulting electronic transitions of LHMHs, whose derived emitters can be employed in anti-counterfeiting and information encryption.

## Abstract 2

The optical activity levels of metal centers govern the multi-mode emissions by manipulating the band alignments in hybrid metal halides.

## Graphical abstract

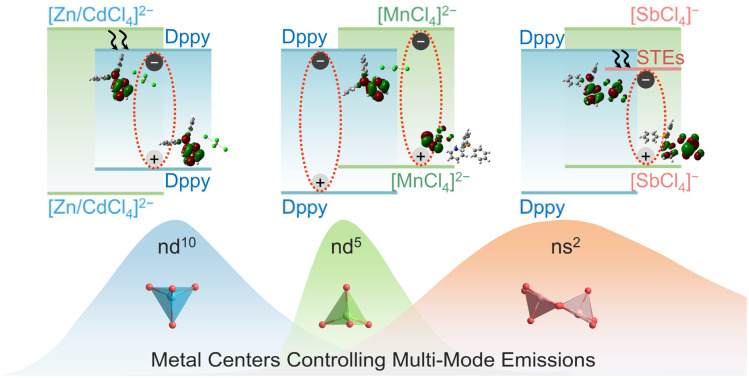

## Introduction

**Scheme 1:**
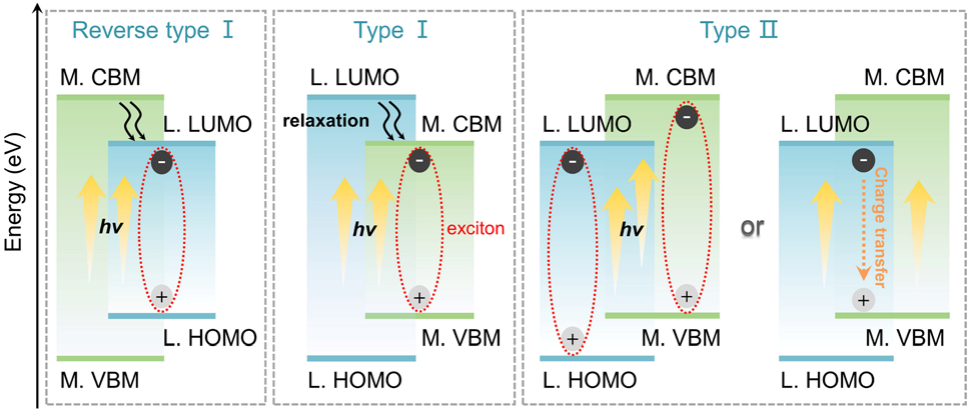
Three types of band alignments in LHMHs (L and M refer to ligands and metals, respectively). Type I (M. CBM–VBM edged), type II (L. LUMO–M. VBM/M. CBM–L. HOMO edged), and reverse type I (L. LUMO–HOMO edged).

Low-dimensional hybrid metal halides (LHMHs) are promising to be an industrialized platform of optical-functional materials in the fields of light-emitting diodes (LEDs)^1–4^ and anti-counterfeiting.^5–8^ This boom is supported by their multi-mode luminescent behaviors, *i.e.*, free exciton (FE) emission,^9–11^ self-trapped exciton (STE) emission,^12–15^ and metal ion (*e.g.*, transition metal ions Mn^2+^, Cr^3+^ or rare-earth ions Eu^3+^, Tb^3+^, *etc.*) transition emission,^16–19^ as well as organic ligand emission.^20–22^ Their resulting electronic transitions strongly depend on the band alignments of LHMHs. As shown in Scheme 1, the band alignment of type I consist of the conduction band minimum (CBM) states of metal cations and the valence band maximum (VBM) states of halogen ions. The highest occupied molecular orbital (HOMO) of organic cations is inferior to the VBM, while the lowest unoccupied molecular orbital (LUMO) of organic cations is superior to the CBM so that the excitons are confined to inorganic fragments and the potential ligand emission is quenched.^23–25^ Type II with a band-edge of LUMO–VBM or CBM–HOMO shows relatively complex photoluminescence (PL) behaviours, involving synergistic emissions as well as charge transfer between organic and inorganic components.^26–28^ In contrast, the band alignment of reverse type I (HOMO–LUMO) allows the excitons to be localized on the organic fragment, thereby facilitating the emission dominated by high-energy excited states of ligands.^29,30^ Although the research on the luminescent properties of LHMHs has achieved periodical progress, there is still limited exploration of the competitive emissions between inorganic units and organic ligands along with the resulting electron transition principles.

Aliphatic organic cations with wide bandgaps normally act as charge balancers and isolate inorganic units in LHMHs, without contributing to the band-edges, tending to construct the band alignment of type I.^31–33^ Conversely, the electronic states of narrow bandgap organic cations with conjugated configurations can extend to the band-edges and form different band alignments. Lü's group has achieved the above three types of band alignments and the conversion between organic and inorganic emissions by incorporating organic cations with varying conjugation degrees with the [PbI_4_]^2−^ inorganic unit. This highlights the crucial role of ligands in the band-edge manipulation and multi-mode emissions of LHMHs.^34^ To date, the dominant metal centers include the cations of Pb^2+^, Sb^3+^, Bi^3+^ and Te^4+^ with ns^2^ electron configuration, the cations of Cu^+^, Cd^2+^, Zn^2+^ and In^3+^ with nd^10^ electron configuration, and the cation of Mn^2+^ with 3d^5^ electron configuration.^35^ The majority of the reported ns^2^-based LHMHs present type I band alignment, with the s orbital of metal cations occupying the VBM and the p orbitals of metal and halogen occupying the CBM, resulting in STE emission from the inorganic units.^36–38^ By contrast, the nd^10^-based LHMHs not only form the type I band alignment composed of the p orbital of halogen as well as s and d orbitals of metal cations but also form type II and reverse type I band alignment involving π and π* orbitals of organic cations, corresponding to the STE emissions of inorganic units, the organic ligand emissions as well as the synergistic emissions of organic and inorganic components.^20,39,40^ Besides, the prevailing Mn^2+^-based (3d^5^) LHMHs typically exhibit the d–d transitions (^4^T_1_ → ^6^A_1_) of Mn^2+^.^41,42^ Neverthless, the working mechanism of metal centers to control multi-mode emissions remains ambiguous, especially when coupled to ligand emission.

Herein, we screened the conjugated aromatic ligand diphenyl-2-pyridylphosphine (Dppy) for assembly with inorganic units [MCl_4_]^*n*−^ (M = Zn^2+^, Cd^2+^, Mn^2+^, and Sb^3+^) with 3d^10^, 4d^10^, 3d^5^ and 5s^2^ electronic configurations, respectively. (DppyH)_2_MCl_4_ (M = Zn^2+^ and Cd^2+^) display an efficient blue emission from the organic ligand, which is attributed to the reverse type I band alignment induced by the large electronegativity and optical inertness of fully-filled nd^10^ shell electrons. However (DppyH)_2_MnCl_4_ with semi-fully filled 3d^5^ shell electrons constructs the band alignment of type II and exhibits the double-peaked emissions from [MnCl_4_]^2−^ and DppyH^+^ at low temperatures. Beyond that, the 5s^2^ lone-pair electrons with stronger stereochemical activity promote (DppyH)SbCl_4_ to reverse the band alignment of type II to type I, resulting in the broadband orange STE emission of [SbCl_4_]^−^. This work highlights the crucial role of the optical activity levels of metal centers in controlling the band alignments and multi-mode emissions while revealing the electronic competition principles between inorganic units and organic ligands. Accordingly, the derivative emitters with abundant light-colors are employed in anti-counterfeiting and information encryption.

## Experimental section

### Materials

The raw materials diphenyl-2-pyridylphosphine (C_17_H_14_NP, 98%, Energy Chemical), zinc chloride (ZnCl_2_, 98%, Aladdin), cadmium chloride hemi(pentahydrate) (CdCl_2_·2.5H_2_O, 98%, Aladdin), manganese chloride (MnCl_2_, ≥99%, Aladdin), antimony trichloride (SbCl_3_, 99%, Macklin), hydrochloric acid (HCl, 37%, Aladdin) and ethanol (C_2_H_5_OH, 99.7%, Guangfu) were used without any further purification.

### Preparation of single crystals

Growth of (DppyH)_2_ZnCl_4_ single crystals. Dppy (1 mmol, 0.2633 g) was dissolved in 1.5 mL HCl for protonation. After stirring for 10 min, ZnCl_2_ (0.5 mmol, 0.0682 g) and 3 mL EtOH were added to the above solution as well as heated and stirred at 70 °C to form a clear solution. Then the container was covered and sealed, and the hydrothermal reactor was placed in an oven at 80 °C. The high-quality single crystals were obtained by decreasing the temperature to 25 °C at a rate of 0.5 °C per hour. The synthetic procedures for (DppyH)_2_CdCl_4_, (DppyH)_2_MnCl_4_ and (DppyH)SbCl_4_ are similar to that for (DppyH)_2_ZnCl_4_.

### Characterization

Single-crystal X-ray diffraction (SCXRD) data of Dppy and (DppyH)_2_CdCl_4_ were collected at 293(2) K using an XtaLAB AFC12 X-ray four-circle single crystal diffractometer (Rigaku) equipped with a CCD-detector, graphite monochromator and Mo-Kα radiation source. The SCXRD data of (DppyH)_2_ZnCl_4_, (DppyH)_2_MnCl_4_ and (DppyH)SbCl_4_ were collected using a D8 Venture diffractometer (Bruker) with Mo-Kα radiation (*λ* = 0.71073 Å). Hirshfeld surfaces and the corresponding 2D fingerprint plots of [MX_4_]^2−^ metal halide anions were calculated using the Crystal Explorer 21.5 program.^43,44^ Powder X-ray diffraction (PXRD) patterns were measured on a Rigaku MiniFlex600 diffractometer with a Cu target tube operating at 40 kV and 15 mA at room temperature (RT) in the 2*θ* range of 5–50°. The morphology analysis and elemental mappings were conducted on a scanning electron microscope (SEM, JEOL JSM-6510). Fourier transform infrared (FTIR) spectra were obtained using an FTIR-650 spectrometer (frequency range from 4000 to 400 cm^−1^) with a KBr pellet. The ultraviolet visible absorption spectroscopy (UV-Vis) was performed on a TU-1901 ultraviolet spectrometer at RT and BaSO_4_ was used as the reflectance standard reference. The PL and photoluminescence excitation (PLE) spectra and photoluminescence quantum yields (PLQYs) were measured using an Edinburgh FLS920 fluorescence spectrometer with a picosecond pulsed diode laser. The temperature-dependent spectra were measured on the Edinburgh FLS980 fluorescence spectrometer equipped with the cryogenic liquid nitrogen plant equipment. PL decay data were collected at RT using an FLS1000 spectrofluorometer using a microsecond light source.

### Computational methods

Density functional theory (DFT) calculations were carried out using the Vienna *ab initio* simulation package code (VASP).^45,46^ The Heyd–Scuseria–Ernzerhof (HSE06)^47^ was applied to calculate the bandgap and electronic properties. A cutoff energy of 400 eV was used for the plane-wave basis set in structural optimizations and electronic structure calculations. The convergence criterion was the energy difference between two consecutive steps of the calculations less than 10^−6^ eV. Besides, the Gaussian 09 program^48^ was used to simulate the HOMO and LUMO distributions.

## Results and discussion

### Structure design and characterization

**Fig. 1:**
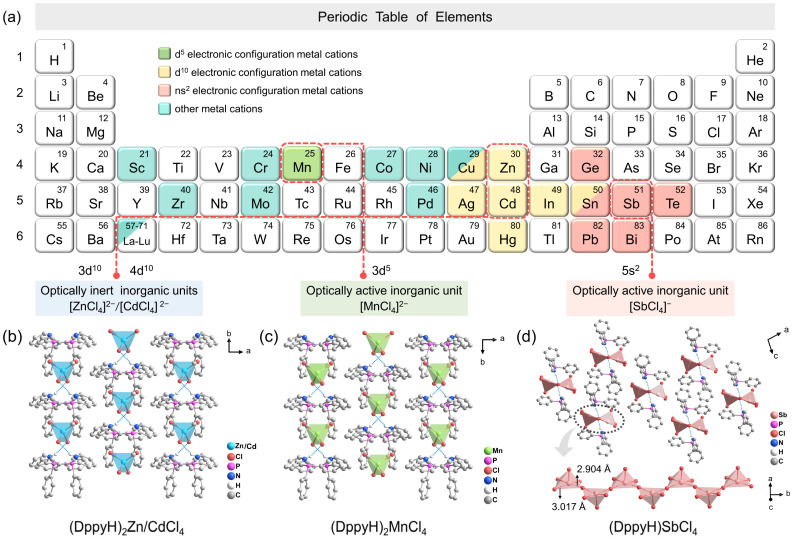
(a) Periodic table of elements. (b and c) The packing diagrams of the crystal structures of (DppyH)_2_MCl_4_ (M = Zn^2+^, Cd^2+^, and Mn^2+^) along the crystallographic *c*-axis. (d) The packing diagram of the crystal structure of (DppyH)SbCl_4_ along the crystallographic *b*-axis, and 1D inorganic infinite chain composed of corner-sharing [SbCl_5_]^2−^ pyramids (the blue dashed lines represent hydrogen bonds and partial hydrogen atoms are omitted).

The aromatic ligand DppyH^+^ was employed as an organic cationic template to assemble with inorganic units [ZnCl_4_]^2−^, [CdCl_4_]^2−^, [MnCl_4_]^2−^ and [SbCl_4_]^−^ with distinct optical activity levels, as shown in Fig. 1a. SCXRD data show that both (DppyH)_2_ZnCl_4_ and (DppyH)_2_CdCl_4_ are isostructural, belonging to the congruent monoclinic space group *C*2/*c*. They display a unique zero-dimensional (0D) isolated structure, in which Zn and Cd atoms are coordinated with four Cl atoms to form individual [MCl_4_]^2−^ twisted tetrahedron units. The organic cations DppyH^+^ separate the inorganic units from each other (Fig. 1b and S1a†), resulting in strong quantum confinement and dielectric confinement effects. The Cd–Cl distances are longer than that of Zn–Cl due to the larger ionic radius of Cd^2+^ than that of Zn^2+^. (DppyH)_2_MnCl_4_ with a 0D structure also belongs to the monoclinic space group *C*2/*c*, but the stacking orientation is opposite to that of (DppyH)_2_MCl_4_ (M = Zn^2+^ and Cd^2+^) (Fig. 1c). By contrast, (DppyH)SbCl_4_ is unusual in that the Sb atom is coordinated to five Cl atoms to form a [SbCl_5_]^2−^ pyramid, which are interconnected by nodes to form an infinitely corrugated one-dimensional (1D) chain along the *b*-axis (Fig. 1d). Among them, the Sb–Cl bond lengths between bridged Cl^−^ and central Sb^3+^ are 2.940 Å and 3.017 Å respectively, which are regarded as Sb⋯Cl secondary bonds (>2.90 Å). Therefore, the inorganic anion chain is actually constructed from multiple [SbCl_3_] trigonal units and free Cl^−^ through Sb⋯Cl secondary bonds.^31^ The Hirshfeld *d*_norm_ surfaces and 2D fingerprint plots point to the intermolecular interactions between organic and inorganic units (Fig. S2–S5†). The crystallographic information files (CIFs) are presented in the ESI,† and the main bond lengths and bond angles are listed in Tables S1–S9.† The FTIR, PXRD and energy dispersive X-ray spectroscopy (EDS) elemental mapping analyses (Fig. S6–S8†) were performed to further verify the phase-purity and associated chemical compositions.

### Optical properties and mechanism investigation

**Fig. 2:**
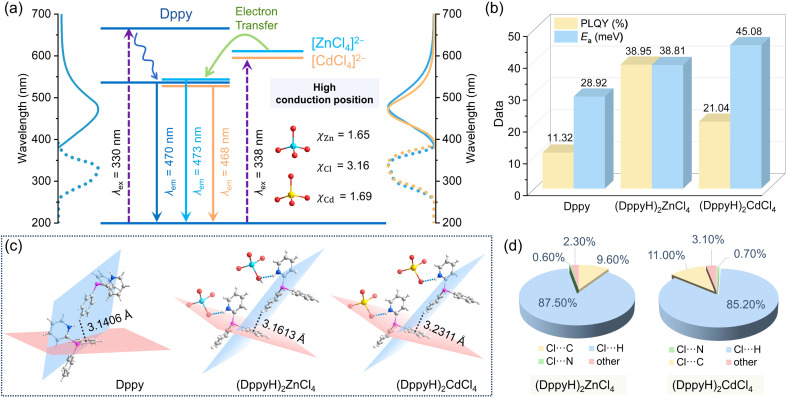
(a) The PLE and PL spectra of Dppy and (DppyH)_2_MCl_4_ (M = Zn^2+^ and Cd^2+^) and the corresponding schematic diagram of the luminescence mechanism. (b) PLQYs and the activation energy *E*_a_ of Dppy and (DppyH)_2_MCl_4_ (M = Zn^2+^ and Cd^2+^). (c) The stacking diagrams of C–H⋯π interaction (black dotted line) between adjacent organic ligands in Dppy and (DppyH)_2_MCl_4_ (M = Zn^2+^ and Cd^2+^) and the N–H⋯Cl interaction (blue dotted line) between the organic cation DppyH^+^ and the inorganic unit [MCl_4_]^2−^ (M = Zn^2+^ and Cd^2+^). (d) Percentage contributions of the various close intermolecular contacts of (DppyH)_2_MCl_4_ (M = Zn^2+^ and Cd^2+^) to the Hirshfeld surface.

The UV-Vis absorption spectra in Fig. S9† indicate that the organic ligand Dppy possesses two absorption peaks at 262 nm and 318 nm, corresponding to the π → π* and n → π* electronic transitions of the benzene and pyridine rings.^27,28^ (DppyH)_2_MCl_4_ (M = Zn^2+^ and Cd^2+^) show similar peak shapes to Dppy, but their absorption bands are broadened, with the bandgaps of 2.95 eV and 2.93 eV, respectively. (DppyH)_2_MnCl_4_ and (DppyH)SbCl_4_ show a wider absorption range from ultraviolet to visible light, as well as narrower bandgaps of 2.85 eV and 2.53 eV, respectively. The optical photograph and CIE chromaticity diagrams are illustrated in Fig. S10.† Subsequently, we have thoroughly investigated their PL properties. As shown in Fig. 2a, Dppy displays a weak blue emission at 470 nm with a full width at half maximum (FWHM) of 98 nm under UV excitation. The PLE and PL spectra in (DppyH)_2_MCl_4_ (M = Zn^2+^ and Cd^2+^) almost overlap with that of Dppy (Fig. 2a). Meanwhile, the PL decay curves show a biexponential decay trend and similar lifetime (Fig. S11†), indicating that the blue emission of (DppyH)_2_MCl_4_ (M = Zn^2+^ and Cd^2+^) is derived from the singlet-state (S_1_ → S_0_) of Dppy. The wavelength-dependent PL and PLE spectra (Fig. S12†) further indicate that the intervention of optically inert units [ZnCl_4_]^2−^ and [CdCl_4_]^2−^ with d^10^ electronic configuration into the optically active ligand cannot modulate the original electronic transition path.

In addition, the inorganic units of [MX_4_]^2−^ (M = Zn^2+^, Cd^2+^ and X = Cl^−^) are discussed as the individual binary discrete system, in which the valence band includes primarily the orbitals of the anion X^−^, while the conduction band includes primarily the orbitals of metal cation M^*n*+^. The energy gap, *E*_g_, involving electron transfer from the valence band to the conduction band, can be qualitatively estimated from the equation:^49^

1 *E*_g_ = 3.72 × (*χ*_anion_ − *χ*_cation_)

where *χ*_anion_ and *χ*_cation_ refer to the optical electronegativity of the anion and cation, respectively. According to Pauling's electronegativity scale,^50^ the electronegativity values of Zn, Cd, and Cl are listed in Fig. 2a. The significant difference in electronegativity between metals and halogen results in a wide bandgap and high conduction band positions in [ZnCl_4_]^2−^ and [CdCl_4_]^2−^. As a result, the binary system is incorporated into the aromatic conjugated ligand with a narrow bandgap, and the ligand primarily occupies the frontier orbitals. Besides, the fully-filled nd^10^(*n* + 1)s^0^ shell electrons exhibit optical inertness. In response to the tetrahedral crystal field, degenerate d orbitals are split into t_2g_ and e_g_ energy levels, and the outer s orbital is split into a_1g_ energy levels. According to the Pauli exclusion principle, the d–d transition is prohibited, and the transfer of excited electrons from the d orbital energy levels to the s orbital energy level (*T*_d_: t_2g_ → a_1g_) requires higher energy, thus preferring the singlet-state (S_1_ → S_0_) emission of Dppy. The corresponding electron transitions are depicted in Fig. 2a. Upon incorporation of the optically inert units [ZnCl_4_]^2−^ and [CdCl_4_]^2−^ into the ligand, the new energy levels are populated in the conduction band of the organic fragment without changing the initial band-edge compositions, causing excited electrons to aggregate at the lowest excitation level of the ligand through non-radiative transitions, thereby facilitating the high-energy emission of Dppy.

Not only that, the introduction of [MCl_4_]^2−^ (M = Zn^2+^ and Cd^2+^) significantly improves the PLQYs (Fig. 2b). The reasons are summarized as follows: (1) the embedding of tetrahedral [ZnCl_4_]^2−^ and [CdCl_4_]^2−^ leads to a larger spacing between the organic ligands (Fig. 2c), thus restraining the self-absorption in the emission centers. (2) The enhanced structural rigidity of (DppyH)_2_MCl_4_ (M = Zn^2+^ and Cd^2+^) suppresses the non-radiative energy loss. The additional hydrogen bonds between the organic cation and inorganic units allow for greater structural stability, with each [MCl_4_]^2−^ unit connecting with six DppyH^+^ cations and each organic cation connecting to three inorganic units to form a robust three-dimensional (3D) hydrogen bonding network (Fig. S13 and S14†). Fig. 2d illustrates that the C/N–H⋯Cl hydrogen bonds play a major role in improving structural rigidity. The temperature-dependent PL spectra and the calculated activation energy *E*_a_ (Fig. S15†) suggest a significant reduction of the thermally activated non-radiation process after the intervention of inorganic units [ZnCl_4_]^2−^ and [CdCl_4_]^2−^, thereby improving the PLQYs.

**Fig. 3:**
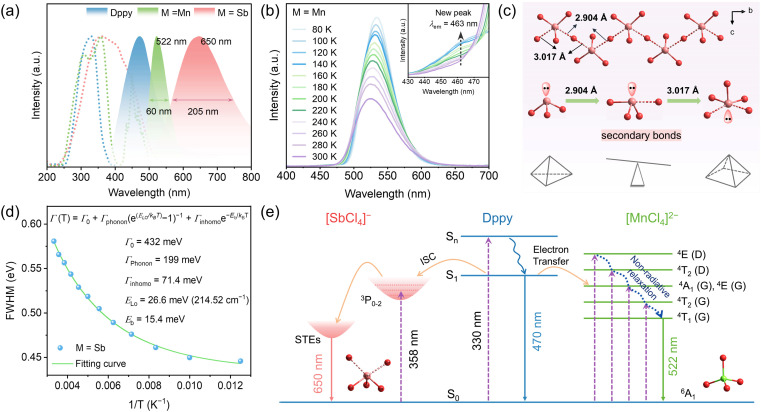
(a) The PLE and PL spectra of Dppy, (DppyH)_2_MnCl_4_ and (DppyH)SbCl_4_. (b) Temperature-dependent PL spectra of (DppyH)_2_MnCl_4_. (c) The evolution process of metal halide units (triangle, seesaw, and pyramid) in (DppyH)SbCl_4_. (d) Bandwidth of the STE emission as a function of 1/T in the range of 80–300 K, in which the green line indicates the fitting data. (e) Schematic diagram of luminescence mechanisms of (DppyH)_2_MnCl_4_ and (DppyH)SbCl_4_.

By contrast, utterly distinct photophysical behaviors were observed in (DppyH)_2_MnCl_4_ and (DppyH)SbCl_4_. As illustrated in Fig. 3a, the PLE spectra show the ultra-broad excitations from the ultraviolet to blue region. Meanwhile, (DppyH)_2_MnCl_4_ exhibits a bright green emission at 522 nm with a narrow FWHM of 60 nm and a PLQY of 50.82%. The PL decay curve (Fig. S16a†) can be fitted with a single-exponential equation: *I*(*t*) = *I*_0_ + *A* exp(−*t*/*τ*), with a lifetime of 2.45 ms, which coincides with the reported hybrid Mn^2+^-based chlorides, demonstrating that the emission belongs to the d–d transition (^4^T_1_ → ^6^A_1_) of Mn^2+^.^51,52^ The wavelength-dependent PLE/PL spectra (Fig. S17a and b†) indicate the existence of a single electron transition pathway and uniform excited state in (DppyH)_2_MnCl_4_ at RT. The temperature-dependent PL spectra (Fig. 3b) indicate that the PL intensity gradually increases as the temperature decreases, and it is noted that an appended emission peak appears at 463 nm. The PL decay curves at 80 K (Fig. S18†) indicate that the luminescence mechanism at 463 nm is different from the emission at 522 nm from [MnCl_4_]^2−^. The nanosecond level lifetime (0.71 ns) is consistent with that of organic ligands. Considering the significant overlap between the PL spectrum of Dppy and the PLE spectrum of [MnCl_4_]^2−^ (Fig. 3a), it is speculated that there is an energy transfer from DppyH^+^ to [MnCl_4_]^2−^. Consequently, the emission at 463 nm can be attributed to the organic ligand, in turn, (DppyH)_2_MnCl_4_ exhibits a synergistic emission of DppyH^+^ and [MnCl_4_]^2−^ at low temperatures. (DppyH)SbCl_4_ exhibits a bright orange emission (PLQY = 37.96%) with a large FWHM of 205 nm and a large Stokes shift of 290 nm, implying that the intervention of optically active unit [SbCl_4_]^−^ can modify or even eliminate the original emission channels. The PL decay data (Fig. S16b†) were characterized as biexponential with an average value of 1.65 ns, which is consistent with the triplet-state STE emission (^3^P_1_ → ^1^S_0_) of LHMHs (Table S10†). Moreover, the wavelength-dependent PLE/PL spectra indicate that there is only the intrinsic emissions from the active units [SbCl_4_]^−^ (Fig. S17c and d†).

The temperature-dependent spectra of (DppyH)SbCl_4_ (Fig. S19†) were recorded to further validate the luminescence mechanism. The relationship curves between bandwidth (*Γ*) and temperature are fitted through the following equation:^53^

2 *Γ*(*T*) = *Γ*_0_ + *Γ*_phonon_(e^(*E*_LO_/*k*_B_T)^ − 1)^−1^ + *Γ*_inhomo_e^−*E*_b_/*k*_B_*T*^

where *Γ*_0_ represents the FWHM at 0 K, and *Γ*_phonon_ and *Γ*_inhomo_ refer to the relative contributions of electron–phonon coupling and trapped states to peaks broadening, respectively. *E*_LO_ is the energy of the longitudinal optical phonon energy and *E*_b_ represents the binding energy of trapped states. The fitting *Γ*_phonon_ is 199 meV, indicating a strong electron–phonon coupling in DppyHSbCl_4_ and a larger contribution of electron–phonon coupling to the PL linewidths than the non-uniform broadening effect (*Γ*_inhomo_). The calculated phonon value (*E*_LO_) is 26.6 meV, which is consistent with the stretching vibration value (215 cm^−1^) of bridging Sb–Cl bonds, validating that STEs are located at the distorted [SbCl_5_]^2−^ pyramid, especially on the bridging Sb–Cl bonds.^54^ Also, the Sb⋯Cl secondary bonds reduce the symmetry of inorganic units, transforming the [SbCl_3_] triangle into an extremely twisted [SbCl_5_]^2−^ pyramid (Fig. 3c). This amplifies the distortion degree in the geometric structures and provides a soft lattice for the formation of STEs. The bond length distortion (Δ*d*)^55^ and bond angle variance (*σ*^2^)^56^ were estimated by using formulae (3) and (4), respectively.3
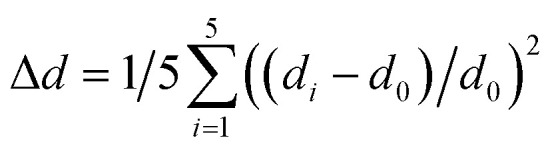
where *d*_0_ represents the average Sb–Cl bond length and *d*_*i*_ are five individual lengths of the Sb–Cl bond.4
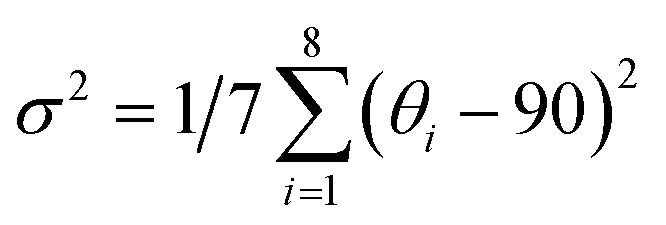
where *θ*_*i*_ refers to all Cl–Sb–Cl bond angles. The Δ*d* and *σ*^2^ values of (DppyH)SbCl_4_ are 1.182 × 10^−2^ and 26.56, respectively, demonstrating a high distortion degree. Furthermore, the weak interaction between Sb and Cl also contributes to the structural distortion and reorganization of excited states.^57,58^

Accordingly, the PL mechanism of (DppyH)_2_MnCl_4_ and (DppyH)SbCl_4_ is illustrated in Fig. 3e. In the case of (DppyH)_2_MnCl_4_, all excited electrons move to the lowest excitation level ^4^T_1_ of [MnCl_4_]^2−^*via* non-radiative relaxation or energy transfer at RT, and then return to the ground state to generate characteristic emission of Mn^2+^. The rate of energy transfer from DppyH^+^ to [MnCl_4_]^2−^ is slowed down with decreasing temperature; most excited electrons follow the transition path as at RT to produce green emission, while a few excited electrons directly return from the excited state of the organic cation to the ground state to emit the singlet-state fluorescence (S_1_ → S_0_), resulting in the dual emissions of DppyH^+^ and [MnCl_4_]^2−^ at 80 K. For (DppyH)SbCl_4_, the excited electrons transfer to the triplet-state levels (^3^P_*n*_) of [SbCl_4_]^−^*via* non-radiative relaxation, where the electrons are captured by phonons to form triplet-state STEs, accompanied by the orange emission while quenching the emission of Dppy.

### Theoretical calculation

**Fig. 4:**
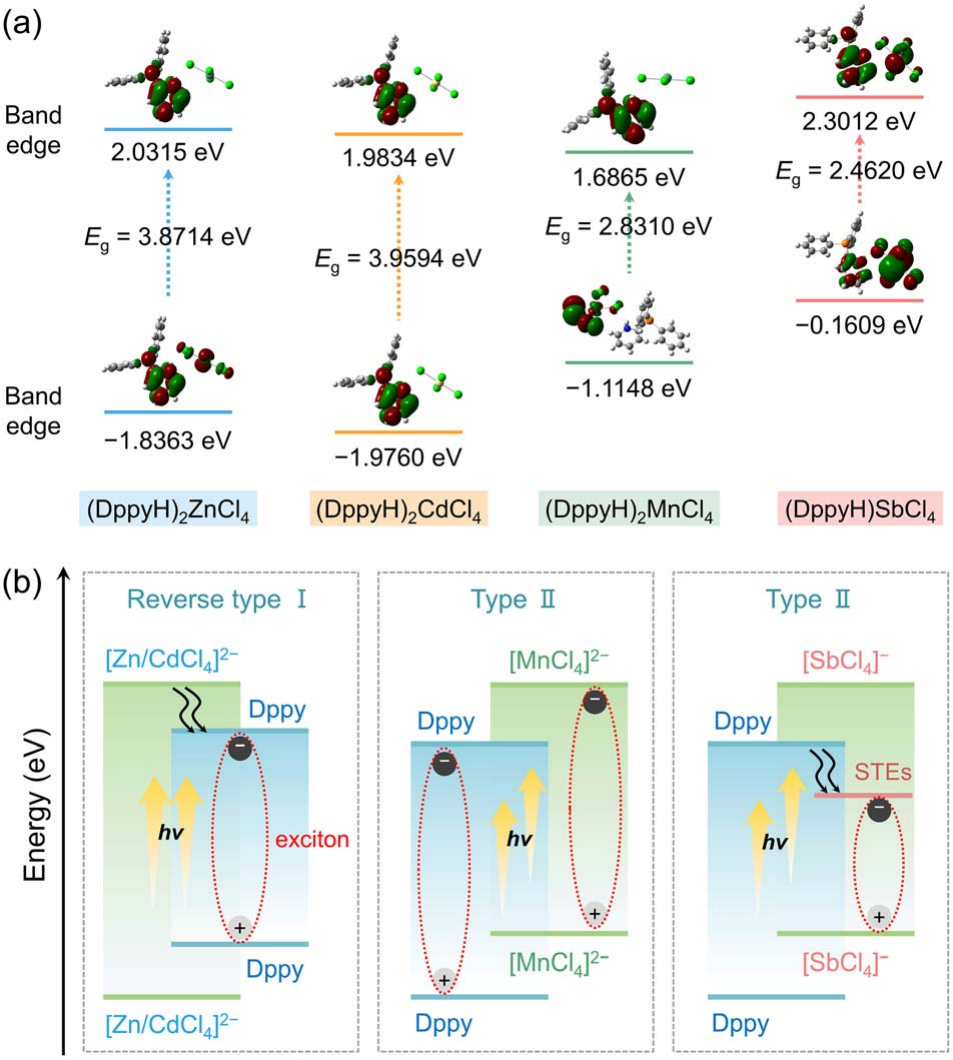
(a and b) Orbital energy levels and corresponding electronic charge densities for the VBM and CBM as well as band alignments of (DppyH)_*n*_MCl_4_ (M = Zn^2+^, Cd^2+^, Mn^2+^, and Sb^3+^).

The DFT calculation was conducted to prove the electron transition mechanism. Fig. 4a and S20† show that the regions from −1.8363/−1.9760 eV to 2.0315/1.9834 eV are determined by the p orbital of C, N and P atoms of the organic ligand in (DppyH)_2_ZnCl_4_ and (DppyH)_2_CdCl_4_. They form the band alignment of reverse type I (Fig. 4b), where the orbitals of DppyH^+^ make the dominant contributions to the VBM states and CBM states, while the orbitals of inorganic units [ZnCl_4_]^2−^ and [CdCl_4_]^2−^ are away from the band-edges, allowing the optical behaviors to be dominated by the electronic transition of the organic ligand. However, the VBM is dominated by the p orbital of Cl in the inorganic anion [MnCl_4_]^2−^, the CBM is occupied by the p orbital of C, N, and P atoms, and the d orbital of Mn appears in the gap of CBM near the band-edge, as for the (DppyH)_2_MnCl_4_, which is aligned with the band alignment of type II. This favors the synergistic emission of DppyH^+^ and [MnCl_4_]^2−^ at low temperatures. In the case of (DppyH)SbCl_4_, the optically active unit [SbCl_4_]^−^ promotes a narrow bandgap. The VBM is dominated by the s and p orbitals of the Sb atom and the p orbital of the Cl atom, with the p orbital of C and P atoms contributing less to the VBM, whereas the p orbital of C and N atoms as well as the s orbital of the Sb atom dominate the CBM. Although (DppyH)SbCl_4_ also forms a band alignment of type II, the temporary defect energy levels induced by 5s^2^ lone-pair electrons promote the band alignment change to type I upon excitation, resulting in the excitons strictly localized in the inorganic unit [SbCl_4_]^−^ and producing the STE emission.^59^ In addition, the calculated optical bandgaps are 3.8714 eV, 3.9594 eV, 2.8310 eV, and 2.4620 eV for (DppyH)_*n*_MCl_4_ (M = Zn^2+^, Cd^2+^, Mn^2+^, and Sb^3+^), respectively (Fig. S21†), which are consistent with the experimental values.

### Anti-counterfeiting application

**Fig. 5:**
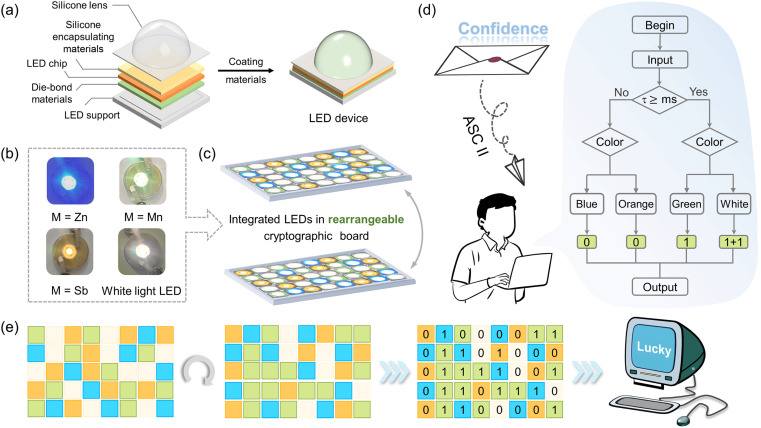
(a) The internal structure and assembly diagram of LED devices. (b) The physical photographs of blue, green, orange and white LED devices at a current of 20 mA, respectively. (c) The model diagram integrating the above four LEDs in a reconfigurable password board. (d) Logic gate application. (e) LED rearrangement and decoding flowchart.

The optical activity levels of metal centers manipulate the band alignments to achieve multi-mode emissions in (DppyH)_*n*_MCl_4_ (M = Zn^2+^, Cd^2+^, Mn^2+^, and Sb^3+^), whose tunable fluorescent color-changing behavior provides a prospective approach for information encryption and transmission. A model instrument incorporating the multicolor UV-converted LED devices with cryptographic devices was engineered in this research. Fig. 5a displays the internal structure and assembly diagram of the LEDs. The blue-emitting (DppyH)_2_ZnCl_4_, green-emitting (DppyH)_2_MnCl_4_, and orange-emitting (DppyH)SbCl_4_ were coated on the UV InGaN chip (365 nm) to acquire the blue, green, and orange UV-converted LED devices, respectively. The white LED device was encapsulated by mixing the above phosphors in a certain proportion. The physical photographs of the UV-converted LED devices at 20 mA current are presented in Fig. 5b. The model device for information encryption and transmission was achieved by integrating four LED devices with a cryptographic board of 5*8 dots, which shuffles and reassembles the LEDs to facilitate the transmission of information with diverse contents (Fig. 5c). As shown in Fig. 5d, we define such a logic gate based on the differences in PL lifetime and colors excited at 365 nm. To enhance the complexity of data encryption, we reference the addition operation: 1 + 1 = 10 (0 carries 1) of the binary system. The output binary codes can be converted into characters through the American Standard Code for Information Interchange (ASC II) to obtain the encrypted information. Fig. 5e illustrates the realignment process of LED devices and the decoding process for a specific LED arranged cryptographic board, culminating in the output of the word “lucky”. The tunable multi-mode emissions in LHMHs are significant for anti-counterfeiting applications.

## Conclusions

In summary, novel LHMHs of (DppyH)_*n*_MCl_4_ (M = Zn^2+^, Cd^2+^, Mn^2+^, and Sb^3+^) were synthesized by incorporating inorganic units with distinct optical activity levels into a highly conjugated organic ligand. (DppyH)_2_MCl_4_ (M = Zn^2+^ and Cd^2+^) exhibit an efficient blue-light emission from the organic ligand due to the formation of the reverse type I band alignment induced by the optical inertness of fully filled d^10^ shell electrons. The PLQYs are significantly improved due to the weakened self-absorption between organic ligands and enhanced structural rigidity after the intervention of [MCl_4_]^2−^ (M = Zn^2+^ and Cd^2+^). By contrast, the optically active [MnCl_4_]^2−^ with semi-full filled 3d^5^ shell electrons constructs the band alignment of type II, resulting in the narrowband green emission from the d–d transition (^4^T_1_ → ^6^A_1_) of Mn^2+^ in (DppyH)_2_MnCl_4_ at RT. (DppyH)SbCl_4_ forms an infinite 1D structure connected by Sb⋯Cl secondary bonds under the strong stereochemical activity of 5s^2^ lone-pair electrons, and the resulting transient defect reverses the band alignment from type II to type I, leading to a bright broadband orange emission from triplet-state (^3^P_1_ → ^1^S_0_) STEs of [SbCl_4_]^−^. The differences in band alignments engineered by optical activity levels of metal centers further reveal the competition mechanism between organic ligands and inorganic units. The derivative emitters with different light-colors present enormous application prospects in the field of anti-counterfeiting and information encryption.

## Author contributions

Qiqiong Ren: prepared and characterised all compounds, conceptualization, investigation, formal analysis, visualization, writing – original draft; Yilin Mao: investigation, formal analysis; Nan Zhang and Jian Zhang: theoretical calculation; Guojun Zhou and Xian-Ming Zhang: conceptualization, project administration, funding acquisition, supervision, writing – review & editing.

## Conflicts of interest

There are no conflicts to declare.

## Supplementary Material

SC-015-D4SC05041J-s001

SC-015-D4SC05041J-s002

## Data Availability

Detailed crystallographic information and supplementary figures and tables can be found in the ESI.† Crystallographic data are available *via* the Cambridge Crystallographic Data Centre (CCDC): 2321320–2321324. The data supporting this article have been included as part of the ESI.†
