# Supplementary material for: Optical activity levels of metal centers controlling multi-mode emissions in low-dimensional hybrid metal halides for anti-counterfeiting and information encryption
Source: Chem Sci. 2024 Sep 16;15(40):16536–45. doi: 10.1039/d4sc05041j (PMC11417954; doi:10.1039/d4sc05041j)
Supplement: SC-015-D4SC05041J-s001 [file SC-015-D4SC05041J-s001.pdf]

Supplementary Information (SI) for Chemical Science.
This journal is © The Royal Society of Chemistry 2024

Supporting Information

Optical Activity Levels of Metal Centers Controlling Multi-Mode Emissions in Low-Dimensional Hybrid Metal Halides for Anti-Counterfeiting and Information Encryption

Qiqiong Ren,[a] Guojun Zhou,*[a] Yilin Mao,[a] Nan Zhang,[a] Jian Zhang,[a] and Xian-Ming Zhang*[ab]

[a] Key Laboratory of Magnetic Molecules and Magnetic Information Materials (Ministry of Education), School of Chemistry and Material Science, Shanxi Normal University, Taiyuan 030031, P. R. China

[b] College of Chemistry & Chemical Engineering, Key Laboratory of Interface Science and Engineering in Advanced Material, Taiyuan University of Technology, Taiyuan, Shanxi 030024, P. R. China

*Corresponding Author(s):

Guojun Zhou: zhougj@sxnu.edu.cn; Xian-Ming Zhang: zhangxm@dns.sxnu.edu.cn

Table of Contents

Table S1. The crystal data of $(DppyH)_nMCl_4$ ($M = Zn^{2+}$, Cd^{2+}, Mn^{2+}, Sb^{3+}).

Table S2-S5. The bond length (Å) of $(DppyH)_nMCl_4$ ($M = Zn^{2+}$, Cd^{2+}, Mn^{2+}, Sb^{3+}).

Table S6-S9. The bond angles (°) of $(DppyH)_nMCl_4$ ($M = Zn^{2+}$, Cd^{2+}, Mn^{2+}, Sb^{3+}).

Table S10. The emission wavelength λ_{em}, FWHM, Huang-Rhys factor S, bond length distortion (Δd) and PL lifetime for the compounds in references.

Fig. S1. Asymmetric units of $(DppyH)_nMCl_4$ ($M = Zn^{2+}$, Cd^{2+}, Mn^{2+}, Sb^{3+}).

Fig. S2-S5. Hirshfeld dnorm surface and 2D fingerprint plots of inorganic units.

Fig. S6. FTIR spectra of $(DppyH)_nMCl_4$ ($M = Zn^{2+}$, Cd^{2+}, Mn^{2+}, Sb^{3+}).

Fig. S7. PXRD of $(DppyH)_nMCl_4$ ($M = Zn^{2+}$, Cd^{2+}, Mn^{2+}, Sb^{3+}).

Fig. S8. SEM images and EDS elemental mapping images of $(DppyH)_nMCl_4$ ($M = Zn^{2+}$, Cd^{2+}, Mn^{2+}, Sb^{3+}).

Fig. S9. UV-vis absorption spectra and the corresponding diffuse reflectance of Dppy and $(DppyH)_nMCl_4$ ($M = Zn^{2+}$, Cd^{2+}, Mn^{2+}, Sb^{3+}).

Fig. S10. Photographs exposed to ambient light and UV excitation and CIE chromaticity diagrams of Dppy and $(DppyH)_nMCl_4$ ($M = Zn^{2+}$, Cd^{2+}, Mn^{2+}, Sb^{3+}).

Fig. S11. PL decay curves of Dppy and $(DppyH)_2MCl_4$ ($M = Zn^{2+}$, Cd^{2+}).

Fig. S12. Wavelength-dependent PL/PLE spectra of $(DppyH)_2MCl_4$ ($M = Zn^{2+}$, Cd^{2+}).

Fig. S13-S14. Intermolecular hydrogen bonds in $(DppyH)_2MCl_4$ ($M = Zn^{2+}$, Cd^{2+}).

Fig. S15. Temperature-dependent PL spectra and calculated the activation energy as a function of Arrhenius equation of Dppy and $(DppyH)_2MCl_4$ ($M = Zn^{2+}$, Cd^{2+}).

Fig. S16. PL decay curves of $(DppyH)_2MCl_4$ ($M = Mn^{2+}$, Sb^{3+}).

Fig. S17. Wavelength-dependent PL and PLE spectra of $(DppyH)_2MCl_4$ ($M = Mn^{2+}$, Sb^{3+}).

Fig. S18. PL decay curves of $(DppyH)_2MnCl_4$ monitored at 463 nm and 522 nm at low temperature 80 K.

Fig. S19. Temperature-dependent PL spectra of $(DppyH)SbCl_4$.

Fig. S20. The total and partial density of states of $(DppyH)_nMCl_4$ ($M = Zn^{2+}$, Cd^{2+}, Mn^{2+}, Sb^{3+}).

Fig. S21. Band structures of $(DppyH)_nMCl_4$ ($M = Zn^{2+}$, Cd^{2+}, Mn^{2+}, Sb^{3+}).

Results and Discussions

Table S1. The crystal data of $(DppyH)_nMCl_4$ ($M = Zn^{2+}, Cd^{2+}, Mn^{2+}, Sb^{3+}$).

Formula	$(DppyH)_2ZnCl_4$	$(DppyH)_2CdCl_4$	$(DppyH)_2MnCl_4$	$(DppyH)SbCl_4$
Mr	735.71	782.74	725.28	527.82
Crystal system	Monoclinic	Monoclinic	Monoclinic	Monoclinic
Space group	C2/c	C2/c	C2/c	P21/n
Z	4	4	4	4
a/Å	16.3042(12)	16.4372(6)	16.3477(10)	11.7340(7)
b/Å	8.1166(6)	8.2217(3)	8.1633(5)	8.8163(4)
c/Å	26.7689(19)	26.8461(9)	26.8181(16)	20.5680(11)
α/°	90	90	90	90
β/°	96.491(2)	96.244(3)	96.399(2)	100.522(2)
γ/°	90	90	90	90
V/Å^3	3519.7(4)	3606.5(2)	3556.6(4)	2091.99(19)
ρ_{calc}/g cm^{-3}	1.388	1.442	1.354	1.676
μ/ mm^{-1}	1.118	1.016	0.787	1.906
F(000)	1504	1576	1484	1032
R_{int}/R_{sigma}	0.0478/0.0354	0.0527/0.0393	0.0529/0.0250	0.0495/0.0380
Reflections	30348	16589	61296	29866
Data/Para	4396/175	4560/195	4469/195	5210/217
$R_1{}^a$, w$R_2{}^b$[I>2σ(I)]	0.0476/0.1161	0.0367/0.0895	0.0386/0.0888	0.0402/0.0849
$R_1{}^a$, w$R_2{}^b$[all data]	0.0758/0.1272	0.0509/0.0982	0.0608/0.0973	0.0635/0.0942
$\Delta\rho_{max}/\Delta\rho_{min}$/ e Å^{-3}	0.836/-0.433	0.435/-0.536	0.484/-0.377	0.793/-0.893

$^aR_1 = \Sigma||F_o| - |F_c||/\ \Sigma|F_o|$. $^bwR_2 = \{\Sigma(w(F_o{}^2 - F_c{}^2)^2)/\Sigma[w(F_o{}^2)^2]\}^{1/2}$

Table S2. The bond length (Å) of $(DppyH)_2ZnCl_4$.

Atom–Atom	Length/Å	Atom–Atom	Length/Å
Zn(1)-Cl(1)	2.2975(8)	C(12)-H(12)	0.9300
Zn(1)-Cl(1)#1	2.2974(8)	C(12)-C(7)	1.3900
Zn(1)-Cl(2)	2.2351(8)	C(17)-H(17)	0.9300
Zn(1)-Cl(2)#1	2.2350(8)	C(17)-C(16)	1.360(5)
P(1)-C(13)	1.829(3)	C(14)-H(14)	0.9300
P(1)-C(7)	1.8200(17)	C(14)-C(15)	1.373(5)
P(1)-C(1)	1.8271(17)	C(16)-H(16)	0.9300
N(1)-H(1)	0.8600	C(16)-C(15)	1.366(6)
N(1)-C(13)	1.343(4)	C(15)-H(15)	0.9300
N(1)-C(17)	1.346(4)	C(6)-H(6)	0.9300
C(13)-C(14)	1.373(4)	C(6)-C(1)	1.3900
C(8)-H(8)	0.9300	C(6)-C(5)	1.3900
C(8)-C(9)	1.3900	C(1)-C(2)	1.3900
C(8)-C(7)	1.3900	C(2)-C(3)	1.3900
C(9)-H(9)	0.9300	C(2)-H(2)	1.10(5)
C(9)-C(10)	1.3900	C(3)-H(3)	0.9300
C(10)-H(10)	0.9300	C(3)-C(4)	1.3900
C(10)-C(11)	1.3900	C(4)-H(4)	0.9300
C(11)-H(11)	0.9300	C(4)-C(5)	1.3900
C(11)-C(12)	1.3900	C(5)-H(5)	0.9300

Table S3. The bond length (Å) of $(DppyH)_2CdCl_4$.

Atom–Atom	Length/Å	Atom–Atom	Length/Å
Cd(1)-Cl(1)#1	2.4812(7)	C(6)-C(1)	1.368(5)
Cd(1)-Cl(1)	2.4813(7)	C(17)-H(17)	0.9300

Cd(1)-Cl(2)	2.4194(7)	C(17)-C(16)	1.365(5)
Cd(1)-Cl(2)#1	2.4195(7)	C(16)-H(16)	0.9300
P(002)-C(5)	1.823(3)	C(16)-C(15)	1.365(6)
P(002)-C(13)	1.835(3)	C(3)-H(3)	0.9300
P(002)-C(7)	1.833(3)	C(3)-C(2)	1.382(5)
N(1)-H(1)	0.8600	C(15)-H(15)	0.9300
N(1)-C(13)	1.345(3)	C(2)-H(2)	0.9300
N(1)-C(17)	1.333(4)	C(2)-C(1)	1.366(6)
C(5)-C(4)	1.386(4)	C(1)-H(1A)	0.9300
C(5)-C(6)	1.400(4)	C(8)-H(8)	0.9300
C(13)-C(14)	1.378(4)	C(8)-C(9)	1.388(5)
C(4)-H(4)	0.9300	C(9)-H(9)	0.9300
C(4)-C(3)	1.379(4)	C(9)-C(10)	1.328(5)
C(7)-C(8)	1.375(4)	C(10)-H(10)	0.9300
C(7)-C(12)	1.358(5)	C(10)-C(11)	1.346(6)
C(14)-H(14)	0.9300	C(12)-H(12)	0.9300
C(14)-C(15)	1.373(5)	C(12)-C(11)	1.396(6)
C(6)-H(6)	0.9300	C(11)-H(11)	0.9300

Table S4. The bond length (Å) of $(DppyH)_2MnCl_4$.

Atom–Atom	Length/Å	Atom–Atom	Length/Å
Mn(1)-Cl(2)#1	2.3819(6)	C(11)-C(10)	1.356(3)
Mn(1)-Cl(2)	2.3819(6)	C(14)-H(14)	0.9300
Mn(1)-Cl(1)	2.3254(6)	C(14)-C(15)	1.376(4)
Mn(1)-Cl(1)#1	2.3254(6)	C(8)-H(8)	0.9300
P(1)-C(12)	1.820(2)	C(8)-C(9)	1.369(4)
P(1)-C(7)	1.832(2)	C(10)-H(10)	0.9300
P(1)-C(1)	1.829(2)	C(10)-C(9)	1.368(4)

N(1)-H(1)	0.8600	C(16)-H(16)	0.9300
N(1)-C(7)	1.340(2)	C(16)-C(15)	1.367(4)
N(1)-C(11)	1.336(3)	C(9)-H(9)	0.9300
C(12)-C(13)	1.387(3)	C(15)-H(15)	0.9300
C(12)-C(17)	1.385(3)	C(6)-H(6)	0.9300
C(7)-C(8)	1.378(3)	C(6)-C(5)	1.382(3)
C(13)-H(13)	0.9300	C(4)-H(4)	0.9300
C(13)-C(14)	1.376(3)	C(4)-C(5)	1.336(4)
C(1)-C(6)	1.366(3)	C(4)-C(3)	1.337(4)
C(1)-C(2)	1.361(3)	C(5)-H(5)	0.9300
C(17)-H(17)	0.9300	C(2)-H(2)	0.9300
C(17)-C(16)	1.371(3)	C(2)-C(3)	1.390(4)
C(11)-H(11)	0.9300	C(3)-H(3)	0.9300

Table S5. The bond length (Å) of (DppyH)$SbCl_4$.

Atom–Atom	Length/Å	Atom–Atom	Length/Å
Sb(1)-Cl(2)	2.4156(11)	C(1)-C(2)	1.399(5)
Sb(1)-Cl(3)	2.3938(13)	C(1)-C(6)	1.388(5)
Sb(1)-Cl(1)	2.3829(14)	C(16)-H(16)	0.9300
P(1)-C(7)	1.816(3)	C(16)-C(17)	1.359(6)
P(1)-C(13)	1.838(3)	C(2)-H(2)	0.9300
P(1)-C(1)	1.815(4)	C(2)-C(3)	1.385(6)
N(1)-H(1)	0.8600	C(10)-H(10)	0.9300
N(1)-C(13)	1.335(5)	C(10)-C(11)	1.359(7)
N(1)-C(17)	1.339(5)	C(10)-C(9)	1.359(8)
C(7)-C(8)	1.385(5)	C(11)-H(11)	0.9300
C(7)-C(12)	1.375(5)	C(9)-H(9)	0.9300
C(13)-C(14)	1.372(5)	C(6)-H(6)	0.9300

C(14)-H(14)	0.9300	C(6)-C(5)	1.380(6)
C(14)-C(15)	1.378(6)	C(17)-H(17)	0.9300
C(8)-H(8)	0.9300	C(3)-H(3)	0.9300
C(8)-C(9)	1.381(6)	C(3)-C(4)	1.368(7)
C(15)-H(15)	0.9300	C(4)-H(4)	0.9300
C(15)-C(16)	1.356(6)	C(4)-C(5)	1.375(7)
C(12)-H(12)	0.9300	C(5)-H(5)	0.9300
C(12)-C(11)	1.388(6)		

Table S6. The bond angles (°) of $(DppyH)_2ZnCl_4$.

Atom–Atom–Atom	Angle/°	Atom–Atom–Atom	Angle/°
Cl(1)#1-Zn(1)-Cl(1)	106.06(5)	C(12)-C(7)-P(1)	114.51(11)
Cl(2)-Zn(1)-Cl(1)	109.49(3)	C(12)-C(7)-C(8)	120.0000
Cl(2)#1-Zn(1)-Cl(1)#1	109.49(3)	N(1)-C(17)-H(17)	120.1000
Cl(2)#1-Zn(1)-Cl(1)	108.79(3)	N(1)-C(17)-C(16)	119.8(4)
Cl(2)-Zn(1)-Cl(1)#1	108.79(3)	C(16)-C(17)-H(17)	120.1000
Cl(2)#1-Zn(1)-Cl(2)	113.92(6)	C(13)-C(14)-H(14)	119.6000
C(7)-P(1)-C(13)	102.61(11)	C(13)-C(14)-C(15)	120.9(3)
C(7)-P(1)-C(1)	104.74(11)	C(15)-C(14)-H(14)	119.6000
C(1)-P(1)-C(13)	100.64(12)	C(17)-C(16)-H(16)	120.8000
C(13)-N(1)-H(1)	118.1000	C(17)-C(16)-C(15)	118.4(3)
C(13)-N(1)-C(17)	123.9(3)	C(15)-C(16)-H(16)	120.8000
C(17)-N(1)-H(1)	118.1000	C(14)-C(15)-H(15)	119.7000
N(1)-C(13)-P(1)	120.0(2)	C(16)-C(15)-C(14)	120.5(3)

N(1)-C(13)-C(14)	116.6(3)	C(16)-C(15)-H(15)	119.7000
C(14)-C(13)-P(1)	123.0(3)	C(1)-C(6)-H(6)	120.0000
C(9)-C(8)-H(8)	120.0000	C(1)-C(6)-C(5)	120.0000
C(9)-C(8)-C(7)	120.0000	C(5)-C(6)-H(6)	120.0000
C(7)-C(8)-H(8)	120.0000	C(6)-C(1)-P(1)	116.53(12)
C(8)-C(9)-H(9)	120.0000	C(2)-C(1)-P(1)	123.47(12)
C(8)-C(9)-C(10)	120.0000	C(2)-C(1)-C(6)	120.0000
C(10)-C(9)-H(9)	120.0000	C(1)-C(2)-H(2)	117.7 (12)
C(9)-C(10)-H(10)	120.0000	C(3)-C(2)-C(1)	120.9 (10)
C(11)-C(10)-C(9)	120.0000	C(3)-C(2)-H(2)	125(3)
C(11)-C(10)-H(10)	120.0000	C(2)-C(3)-H(3)	120.0000
C(10)-C(11)-H(11)	120.0000	C(4)-C(3)-C(2)	115(3)
C(10)-C(11)-C(12)	120.0000	C(4)-C(3)-H(3)	120.0000
C(12)-C(11)-H(11)	120.0000	C(3)-C(4)-H(4)	120.0000
C(11)-C(12)-H(12)	120.0000	C(3)-C(4)-C(5)	120.0000
C(11)-C(12)-C(7)	120.0000	C(5)-C(4)-H(4)	120.0000
C(7)-C(12)-H(12)	120.0000	C(6)-C(5)-H(5)	120.0000
C(8)-C(7)-P(1)	125.44(11)	C(4)-C(5)-C(6)	120.0000

Table S7. The bond angles (°) of $(DppyH)_2CdCl_4$.

Atom–Atom–Atom	Angle/°	Atom–Atom–Atom	Angle/°
Cl(1)#1-Cd(1)-Cl(1)	103.97(4)	C(16)-C(17)-H(17)	120.0000
Cl(2)-Cd(1)-Cl(1)#1	109.08(3)	C(17)-C(16)-H(16)	120.9000

Cl(2)-Cd(1)-Cl(1)	109.37(3)	C(17)-C(16)-C(15)	118.2(3)
Cl(2)#1-Cd(1)-Cl(1)	109.08(3)	C(15)-C(16)-H(16)	120.9000
Cl(2)#1-Cd(1)-Cl(1)#1	109.37(3)	C(4)-C(3)-H(3)	120.2000
Cl(2)-Cd(1)-Cl(2)#1	115.37(5)	C(4)-C(3)-C(2)	119.6(3)
C(5)-P(002)-C(13)	102.17(12)	C(2)-C(3)-H(3)	120.2000
C(5)-P(002)-C(7)	104.68(13)	C(14)-C(15)-H(15)	119.8000
C(7)-P(002)-C(13)	100.60(12)	C(16)-C(15)-C(14)	120.5(3)
C(13)-N(1)-H(1)	117.9000	C(16)-C(15)-H(15)	119.8000
C(17)-N(1)-H(1)	117.9000	C(3)-C(2)-H(2)	119.9000
C(17)-N(1)-C(13)	124.1(3)	C(1)-C(2)-C(3)	120.2(3)
C(4)-C(5)-P(002)	126.1(2)	C(1)-C(2)-H(2)	119.9000
C(4)-C(5)-C(6)	118.3(3)	C(6)-C(1)-H(1A)	119.8000
C(6)-C(5)-P(002)	115.6(2)	C(2)-C(1)-C(6)	120.5(3)
N(1)-C(13)-P(002)	120.32(19)	C(2)-C(1)-H(1A)	119.8000
N(1)-C(13)-C(14)	116.3(3)	C(7)-C(8)-H(8)	119.7000
C(14)-C(13)-P(002)	123.1(2)	C(7)-C(8)-C(9)	120.7(3)
C(5)-C(4)-H(4)	119.6000	C(9)-C(8)-H(8)	119.7000
C(3)-C(4)-C(5)	120.8(3)	C(8)-C(9)-H(9)	119.5000
C(3)-C(4)-H(4)	119.6000	C(10)-C(9)-C(8)	121.0(4)
C(8)-C(7)-P(002)	124.6(2)	C(10)-C(9)-H(9)	119.5000
C(12)-C(7)-P(002)	117.7(3)	C(9)-C(10)-H(10)	120.3000
C(12)-C(7)-C(8)	117.6(3)	C(9)-C(10)-C(11)	119.4(4)

C(13)-C(14)-H(14)	119.6000	C(11)-C(10)-H(10)	120.3000
C(15)-C(14)-C(13)	120.9(3)	C(7)-C(12)-H(12)	119.7000
C(15)-C(14)-H(14)	119.6000	C(7)-C(12)-C(11)	120.5(4)
C(5)-C(6)-H(6)	119.7000	C(11)-C(12)-H(12)	119.7000
C(1)-C(6)-C(5)	120.6(3)	C(10)-C(11)-C(12)	120.7(4)
C(1)-C(6)-H(6)	119.7000	C(10)-C(11)-H(11)	119.6000
N(1)-C(17)-H(17)	120.0000	C(12)-C(11)-H(11)	119.6000
N(1)-C(17)-C(16)	120.1(3)		

Table S8. The bond angles (°) of $(DppyH)_2MnCl_4$.

Atom–Atom–Atom	Angle/°	Atom–Atom–Atom	Angle/°
Cl(2)#1-Mn(1)-Cl(2)	105.13(3)	C(15)-C(14)-H(14)	120.0000
Cl(1)#1-Mn(1)-Cl(2)#1	109.590(19)	C(7)-C(8)-H(8)	119.6000
Cl(1)-Mn(1)-Cl(2)#1	108.99(2)	C(9)-C(8)-C(7)	120.8(2)
Cl(1)-Mn(1)-Cl(2)	109.590(19)	C(9)-C(8)-H(8)	119.6000
Cl(1)#1-Mn(1)-Cl(2)	108.99(2)	C(11)-C(10)-H(10)	120.9000
Cl(1)-Mn(1)-Cl(1)#1	114.15(4)	C(11)-C(10)-C(9)	118.3(2)
C(12)-P(1)-C(7)	102.34(9)	C(9)-C(10)-H(10)	120.9000
C(12)-P(1)-C(1)	104.71(9)	C(17)-C(16)-H(16)	119.9000
C(1)-P(1)-C(7)	100.50(9)	C(15)-C(16)-C(17)	120.2(2)
C(7)-N(1)-H(1)	118.0000	C(15)-C(16)-H(16)	119.9000
C(11)-N(1)-H(1)	118.0000	C(8)-C(9)-H(9)	119.8000
C(11)-N(1)-C(7)	124.04(18)	C(10)-C(9)-C(8)	120.4(2)

C(13)-C(12)-P(1)	125.82(15)	C(10)-C(9)-H(9)	119.8000
C(17)-C(12)-P(1)	115.65(16)	C(14)-C(15)-H(15)	119.9000
C(17)-C(12)-C(13)	118.5(2)	C(16)-C(15)-C(14)	120.1(2)
N(1)-C(7)-P(1)	120.34(14)	C(16)-C(15)-H(15)	119.9000
N(1)-C(7)-C(8)	116.38(19)	C(1)-C(6)-H(6)	119.5000
C(8)-C(7)-P(1)	122.85(17)	C(1)-C(6)-C(5)	121.1(2)
C(12)-C(13)-H(13)	119.8000	C(5)-C(6)-H(6)	119.5000
C(14)-C(13)-C(12)	120.5(2)	C(5)-C(4)-H(4)	120.3000
C(14)-C(13)-H(13)	119.8000	C(5)-C(4)-C(3)	119.3(3)
C(6)-C(1)-P(1)	124.73(16)	C(3)-C(4)-H(4)	120.3000
C(2)-C(1)-P(1)	117.67(18)	C(6)-C(5)-H(5)	119.7000
C(2)-C(1)-C(6)	117.6(2)	C(4)-C(5)-C(6)	120.6(3)
C(12)-C(17)-H(17)	119.6000	C(4)-C(5)-H(5)	119.7000
C(16)-C(17)-C(12)	120.8(2)	C(1)-C(2)-H(2)	119.9000
C(16)-C(17)-H(17)	119.6000	C(1)-C(2)-C(3)	120.3(3)
N(1)-C(11)-H(11)	120.0000	C(3)-C(2)-H(2)	119.9000
N(1)-C(11)-C(10)	120.1(2)	C(4)-C(3)-C(2)	121.1(3)
C(10)-C(11)-H(11)	120.0000	C(4)-C(3)-H(3)	119.5000
C(13)-C(14)-H(14)	120.0000	C(2)-C(3)-H(3)	119.5000
C(13)-C(14)-C(15)	119.9(2)		

Table S9. The bond angles (°) of $(DppyH)SbCl_4$.

Atom–Atom–Atom	Angle/°	Atom–Atom–Atom	Angle/°
Cl(3)-Sb(1)-Cl(2)	91.20(5)	C(15)-C(16)-H(16)	120.5000
Cl(1)-Sb(1)-Cl(2)	93.30(6)	C(15)-C(16)-C(17)	119.1(4)
Cl(1)-Sb(1)-Cl(3)	99.97(8)	C(17)-C(16)-H(16)	120.5000
C(7)-P(1)-C(13)	103.13(16)	C(1)-C(2)-H(2)	120.0000
C(1)-P(1)-C(7)	105.11(17)	C(3)-C(2)-C(1)	120.0(4)
C(1)-P(1)-C(13)	101.91(16)	C(3)-C(2)-H(2)	120.0000
C(13)-N(1)-H(1)	118.2000	C(11)-C(10)-H(10)	119.6000
C(13)-N(1)-C(17)	123.5(4)	C(11)-C(10)-C(9)	120.8(4)
C(17)-N(1)-H(1)	118.2000	C(9)-C(10)-H(10)	119.6000
C(8)-C(7)-P(1)	115.7(3)	C(12)-C(11)-H(11)	119.9000
C(12)-C(7)-P(1)	124.6(3)	C(10)-C(11)-C(12)	120.2(5)
C(12)-C(7)-C(8)	119.7(4)	C(10)-C(11)-H(11)	119.9000
N(1)-C(13)-P(1)	116.1(3)	C(8)-C(9)-H(9)	120.0000
N(1)-C(13)-C(14)	117.4(3)	C(10)-C(9)-C(8)	120.1(5)
C(14)-C(13)-P(1)	126.1(3)	C(10)-C(9)-H(9)	120.0000
C(13)-C(14)-H(14)	119.9000	C(1)-C(6)-H(6)	119.7000
C(13)-C(14)-C(15)	120.2(4)	C(5)-C(6)-C(1)	120.7(4)
C(15)-C(14)-H(14)	119.9000	C(5)-C(6)-H(6)	119.7000
C(7)-C(8)-H(8)	120.1000	N(1)-C(17)-C(16)	119.6(4)
C(9)-C(8)-C(7)	119.7(4)	N(1)-C(17)-H(17)	120.2000
C(9)-C(8)-H(8)	120.1000	C(16)-C(17)-H(17)	120.2000

C(14)-C(15)-H(15)	119.9000	C(2)-C(3)-H(3)	119.8000
C(16)-C(15)-C(14)	120.2(4)	C(4)-C(3)-C(2)	120.4(4)
C(16)-C(15)-H(15)	119.9000	C(3)-C(4)-H(4)	119.9000
C(7)-C(12)-H(12)	120.2000	C(3)-C(4)-C(5)	120.3(4)
C(7)-C(12)-C(11)	119.6(4)	C(5)-C(4)-H(4)	119.9000
C(11)-C(12)-H(12)	120.2000	C(6)-C(5)-H(5)	120.0000
C(2)-C(1)-P(1)	117.4(3)	C(4)-C(5)-C(6)	120.0(4)
C(6)-C(1)-P(1)	123.9(3)	C(4)-C(5)-H(5)	120.0000
C(6)-C(1)-C(2)	118.6(4)		

Table S10. The emission wavelength λ_{em}, FWHM, Huang-Rhys factor *S*, bond length distortion (Δd) and PL lifetime for the compounds in references.

Compound	λ_{em} (nm)	FWHM (nm)	S	Δd	τ (ns)	reference
$(TMEDA)_3Sb_2Br_{12}\cdot H_2O$	625	150	21.2	–	34.7	[1]
$(PMA)_3BiBr_6$	510	153	13.7	2.10×10^{-4}	1.031	[2]
$(PMA)_3SbBr_6$	625	175	12.5	7.10×10^{-4}	1.508	[2]
$(DppyH)SbCl_4$	650	205	19.45	1.182×10^{-2}	2.07	**this work**

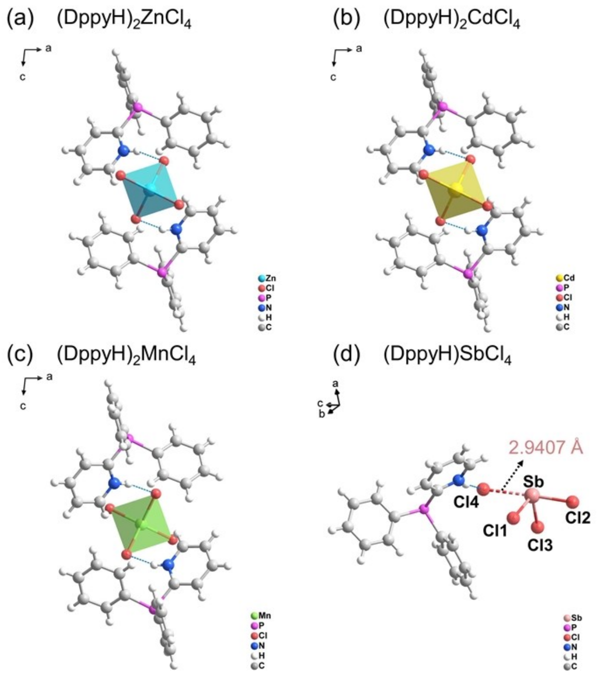

Fig. S1. Asymmetric units of $(DppyH)_nMCl_4$ (M = Zn^{2+}, Cd^{2+}, Mn^{2+}, Sb^{3+}).

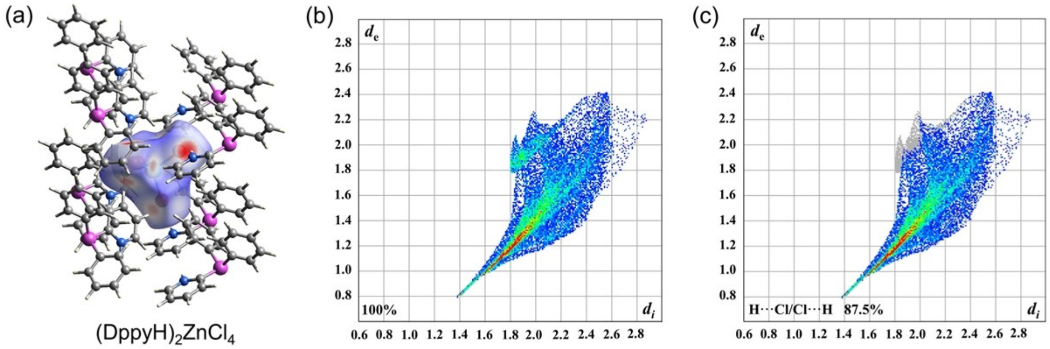

Fig. S2. (a) Hirshfeld dnorm surface and (b, c) 2D fingerprint plots of $[ZnCl_4]^{2-}$.

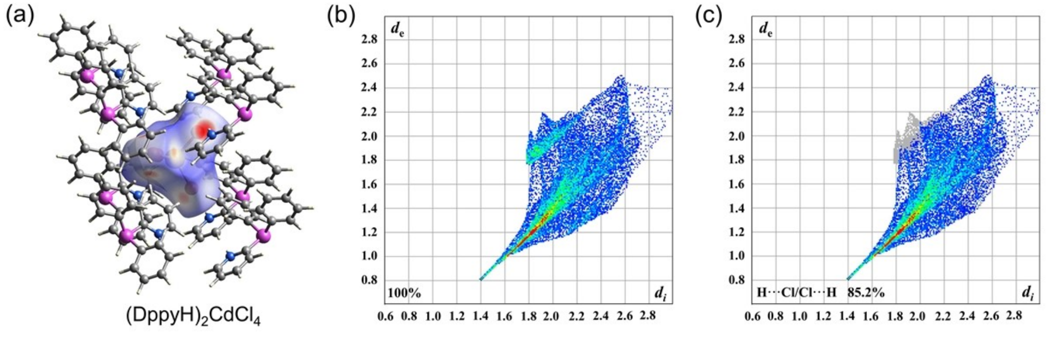

Fig. S3. (a) Hirshfeld dnorm surface and (b, c) 2D fingerprint plots of $[CdCl_4]^{2-}$.

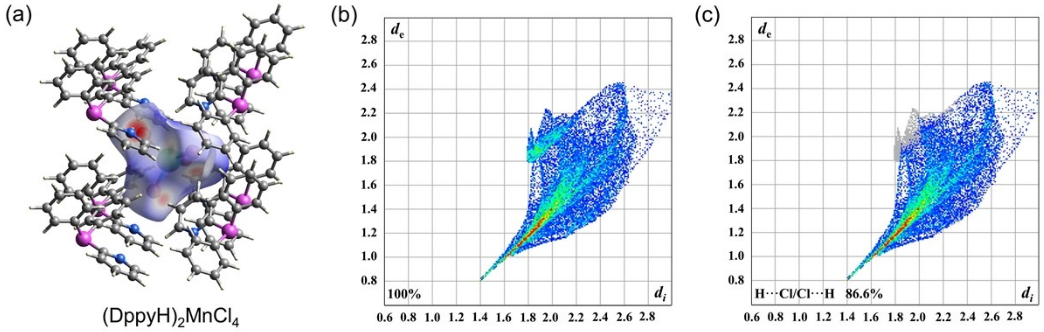

Fig. S4. (a) Hirshfeld dnorm surface and (b, c) 2D fingerprint plots of $[MnCl_4]^{2-}$.

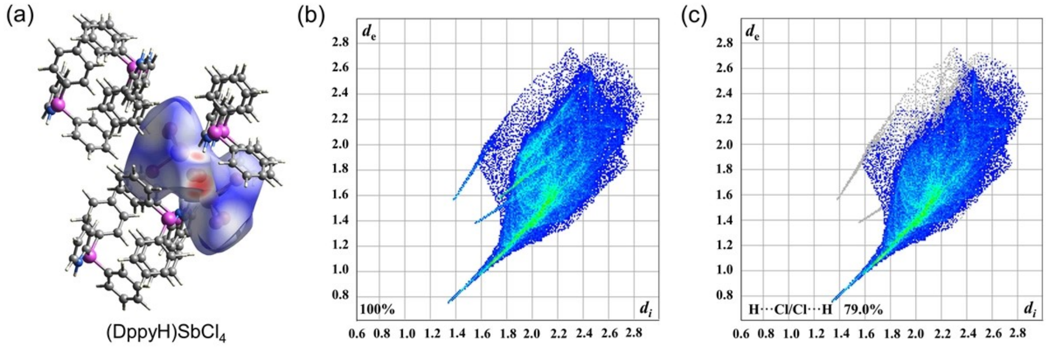

Fig. S5. (a) Hirshfeld dnorm surface and (b, c) 2D fingerprint plots of $[SbCl_4]^{-}$.

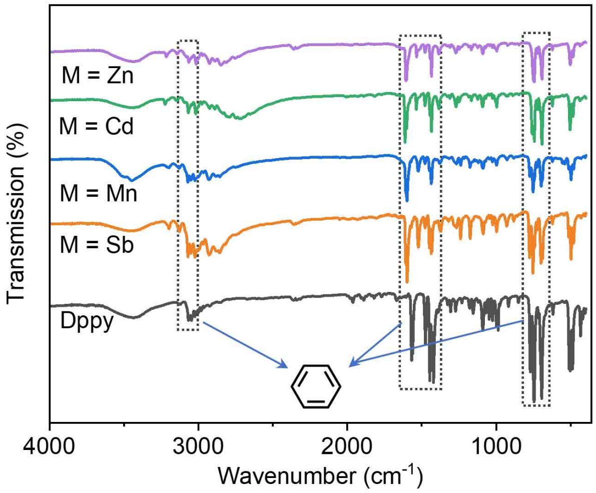

Fig. S6. FTIR spectra of $(DppyH)_nMCl_4$ ($M = Zn^{2+}$, Cd^{2+}, Mn^{2+}, Sb^{3+}).

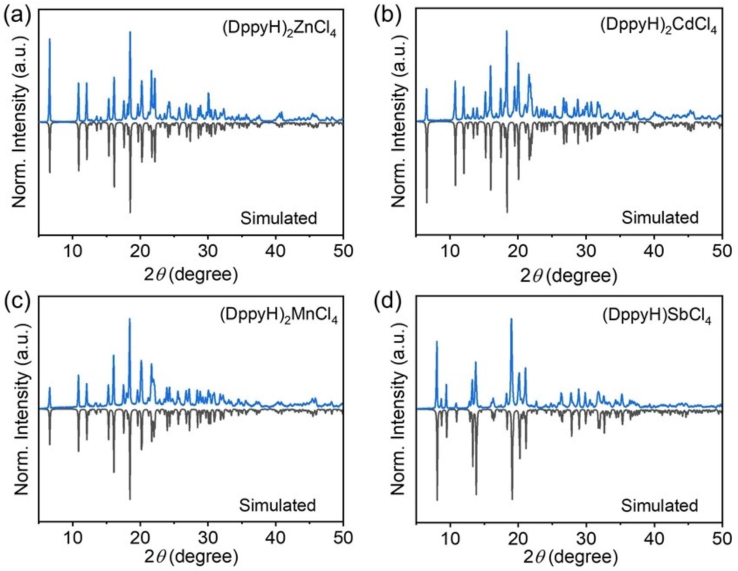

Fig. S7. (a-d) PXRD of $(DppyH)_nMCl_4$ ($M = Zn^{2+}, Cd^{2+}, Mn^{2+}, Sb^{3+}$).

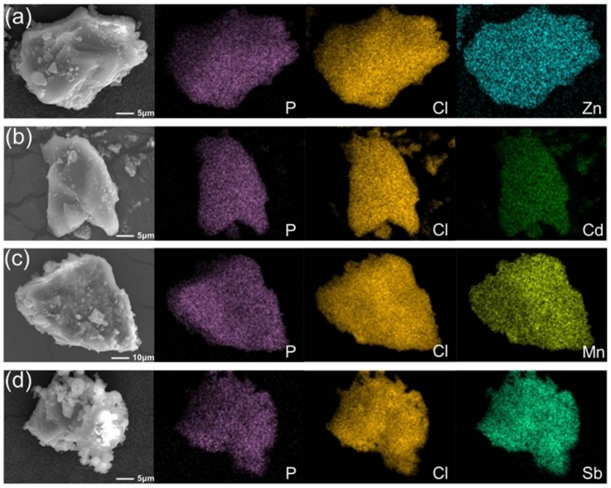

Fig. S8. (a-d) SEM images and EDS elemental mapping images of powder samples of $(DppyH)_nMCl_4$ ($M = Zn^{2+}, Cd^{2+}, Mn^{2+}, Sb^{3+}$).

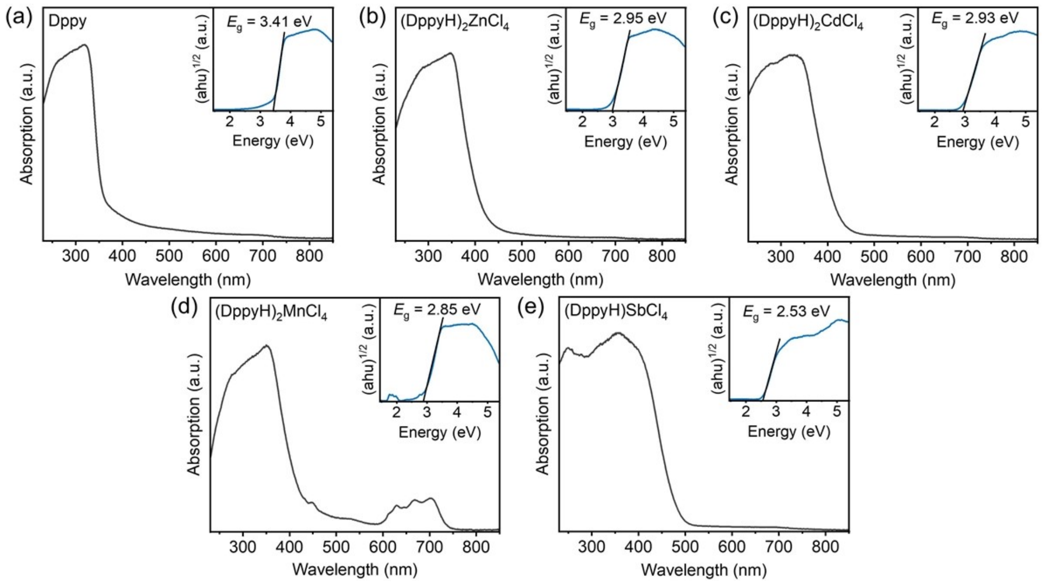

Fig. S9. (a-e) UV-vis absorption spectra and the corresponding diffuse reflectance of Dppy and $(DppyH)_nMCl_4$ (M = Zn^{2+}, Cd^{2+}, Mn^{2+}, Sb^{3+}).

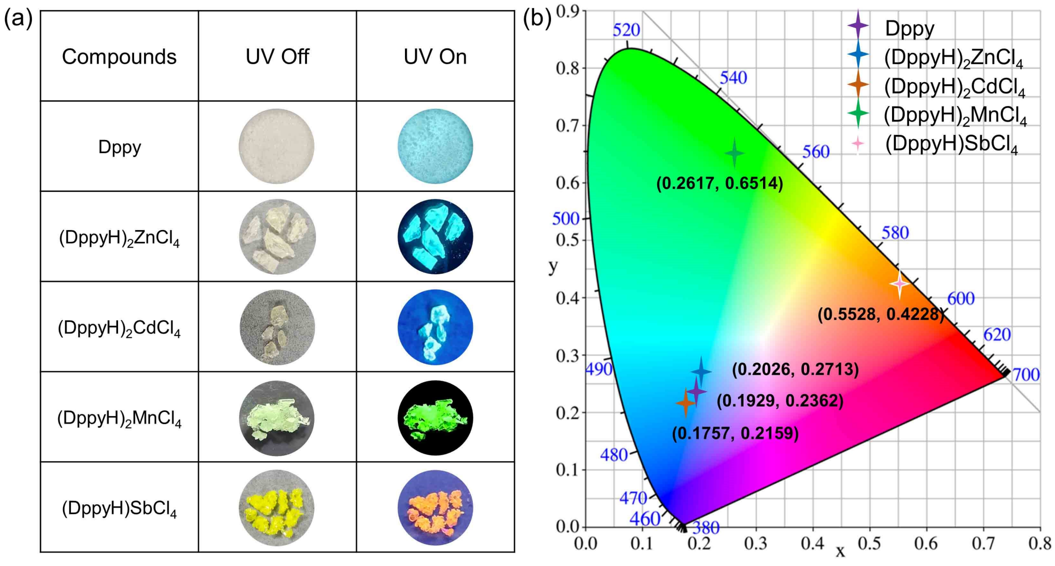

Fig. S10. (a, b) The photographs exposed to ambient light and UV excitation and CIE chromaticity diagrams of Dppy and $(DppyH)_nMCl_4$ (M = Zn^{2+}, Cd^{2+}, Mn^{2+}, Sb^{3+}).

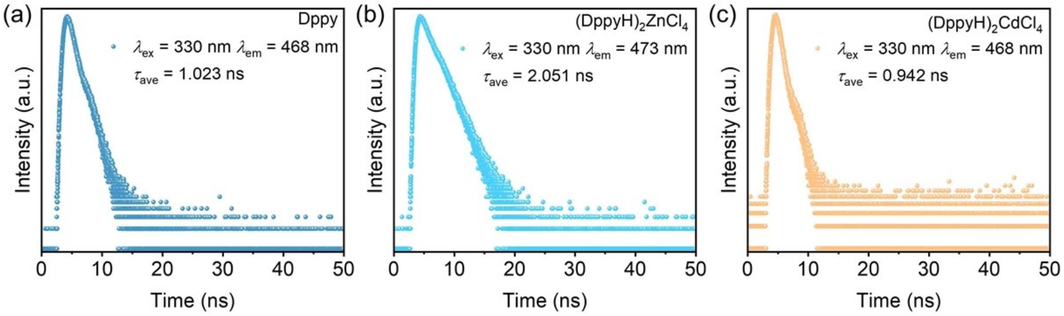

Fig. S11. (a-c) The PL decay curves of Dppy and $(DppyH)_2MCl_4$ ($M = Zn^{2+}$, Cd^{2+}).

The biexponential PL decay curves can be well fitted by using the following decay function:[3]

$$I(t) = A_1 \, exp\,(-t/\tau_1) + A_2 \, exp\,(-t/\tau_2) \tag{3}$$

The average lifetime is calculated by formula:[4]

$$\tau_{ave} = (A_1\tau_1^2 + A_2\tau_2^2)/(A_1\tau_1 + A_2\tau_2) \tag{4}$$

where τ_1 and τ_2 are the PL lifetimes, and A_1 and A_2 are the coefficients of the exponential decay terms. The average lifetimes of Dppy and $(DppyH)_2MCl_4$ ($M = Zn^{2+}$, Cd^{2+}) can be attributed to the nanosecond levels at 1.023 ns, 2.051 ns and 0.942 ns, respectively.

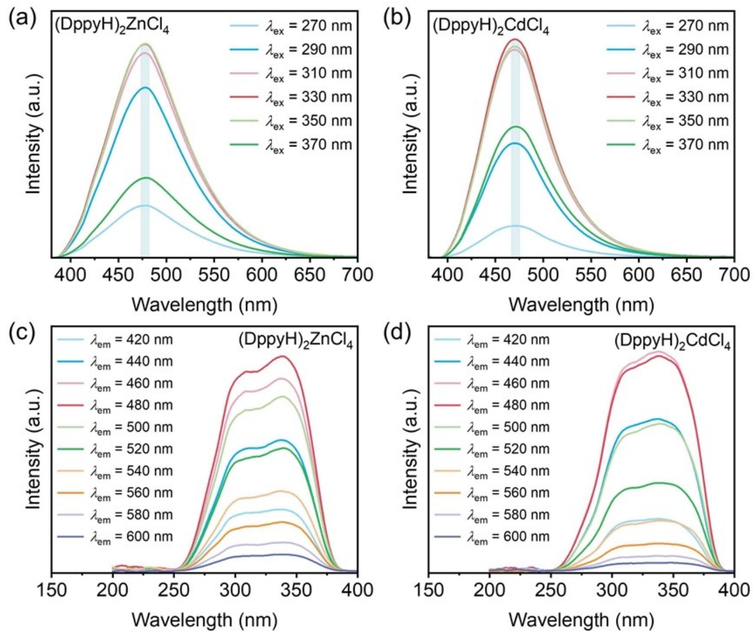

Fig. S12. (a, b) Wavelength-dependent PL spectra and (c, d) Wavelength-dependent PLE spectra of $(DppyH)_2MCl_4$ ($M = Zn^{2+}$, Cd^{2+}).

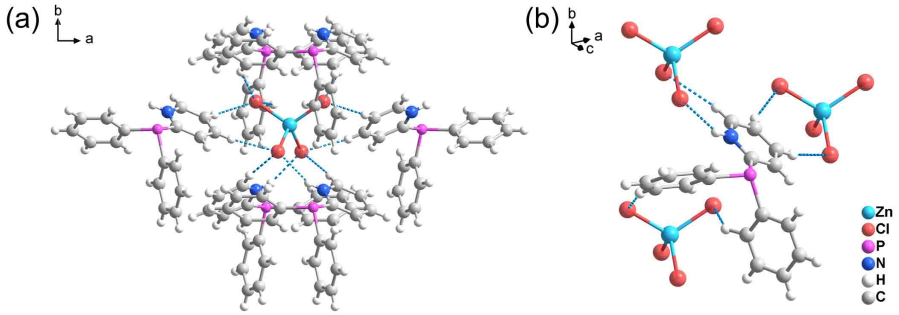

Fig. S13. Intermolecular Hydrogen bonds in $(DppyH)_2ZnCl_4$. (a) Each $[ZnCl_4]^{2-}$ anion connects with six $(DppyH)^+$ organic cations. (b) Each $(DppyH)^+$ organic cation link with three anions.

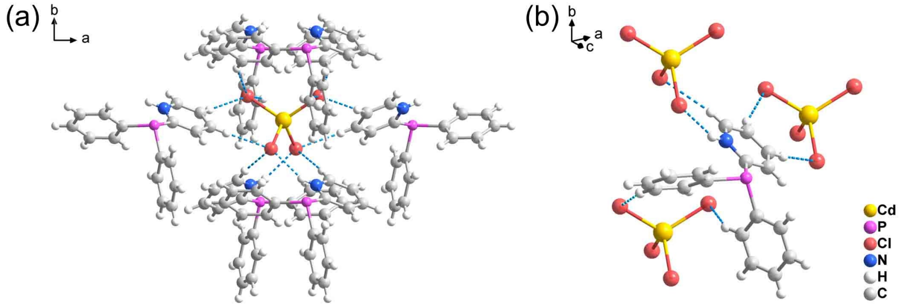

Fig. S14. Intermolecular Hydrogen bonds in $(DppyH)_2CdCl_4$. (a) Each $[CdCl_4]^{2-}$ anion connects with six $(DppyH)^+$ organic cations. (b) Each $(DppyH)^+$ organic cation link with three anions.

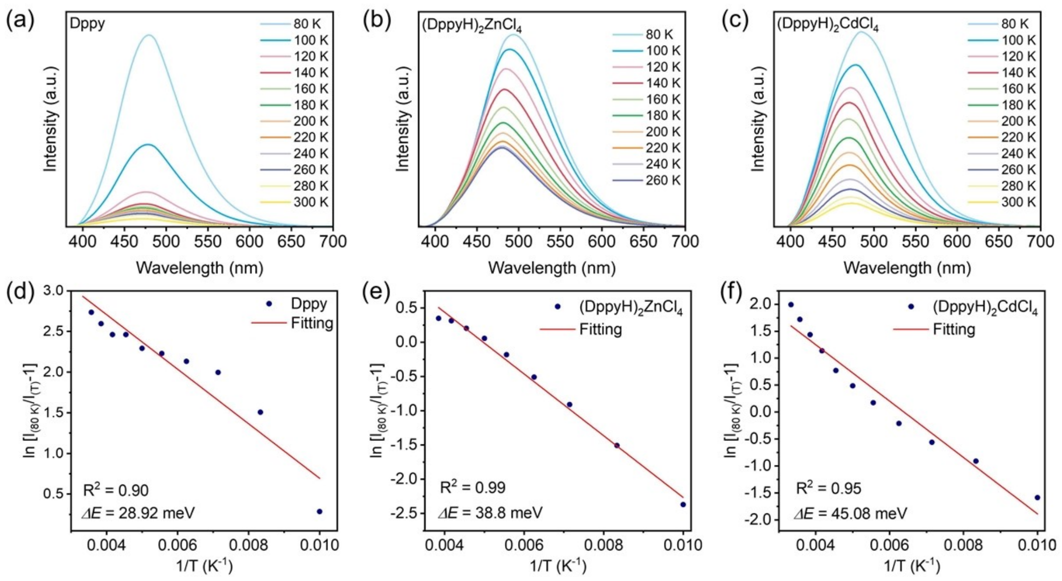

Fig. S15. (a-c) Temperature-dependent PL spectra and (d-f) Calculated the activation energy as a function of Arrhenius equation of Dppy and $(DppyH)_2MCl_4$ ($M = Zn^{2+}$, Cd^{2+}).

The thermally activated nonradiative recombination can be described by the following Arrhenius equation:[5]

$$I(T) = \frac{I_0}{1 + A\,exp\,(-\frac{E_a}{kT})} \qquad (4)$$

where I_0 is the PL intensity at 80 K, $I(T)$ is the PL intensity at any temperature, A refers to the pre-exponential factor, k is the Boltzmann constant, and E_a is the activation energy. The activation energies are calculated as 28.92 meV, 38.81 meV, and 45.08 meV for Dppy and $(DppyH)_2MCl_4$ (M = Zn^{2+}, Cd^{2+}), respectively.

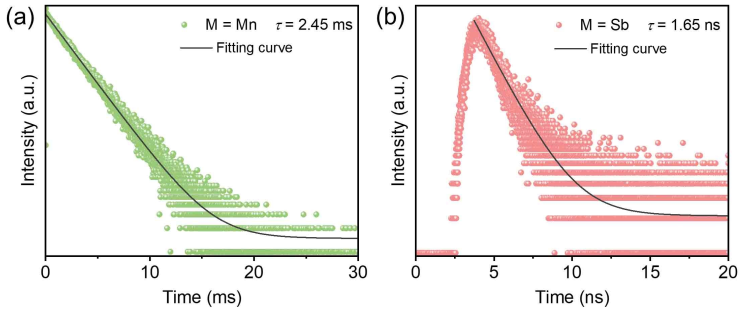

Fig. S16. (a, b) PL decay curves of $(DppyH)_2MCl_4$ ($M = Mn^{2+}$, Sb^{3+}) at room temperature.

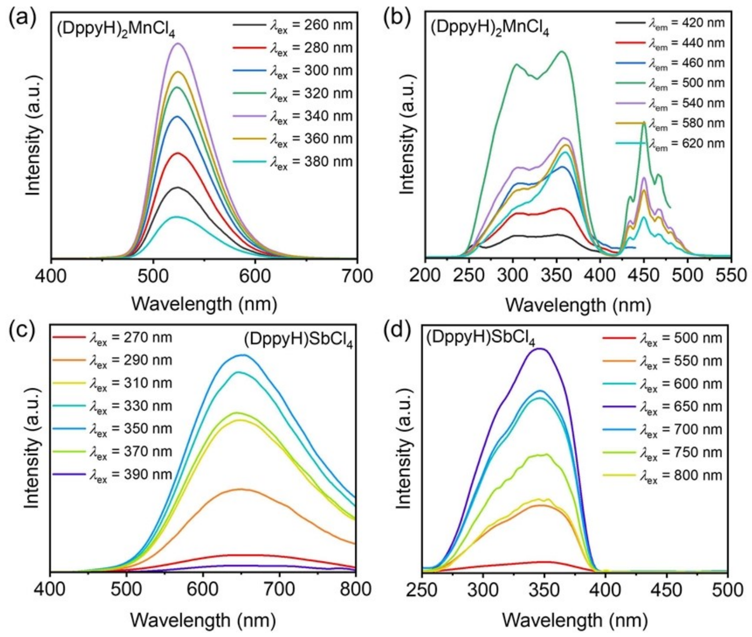

Fig. S17. (a-d) Wavelength-dependent PL and PLE spectra of $(DppyH)_2MCl_4$ ($M = Mn^{2+}$, Sb^{3+}).

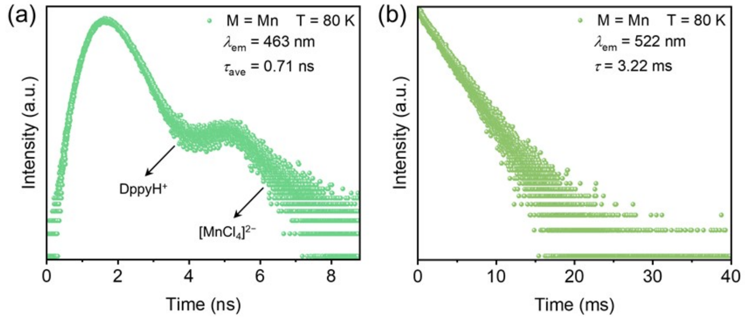

Fig. S18. (a, b) PL decay curves of $(DppyH)_2MnCl_4$ monitored at 463 nm and 522 nm at low temperature 80 K.

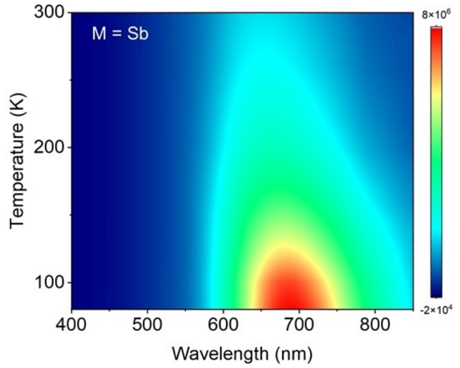

Fig. S19. Temperature-dependent PL spectra of $(DppyH)SbCl_4$.

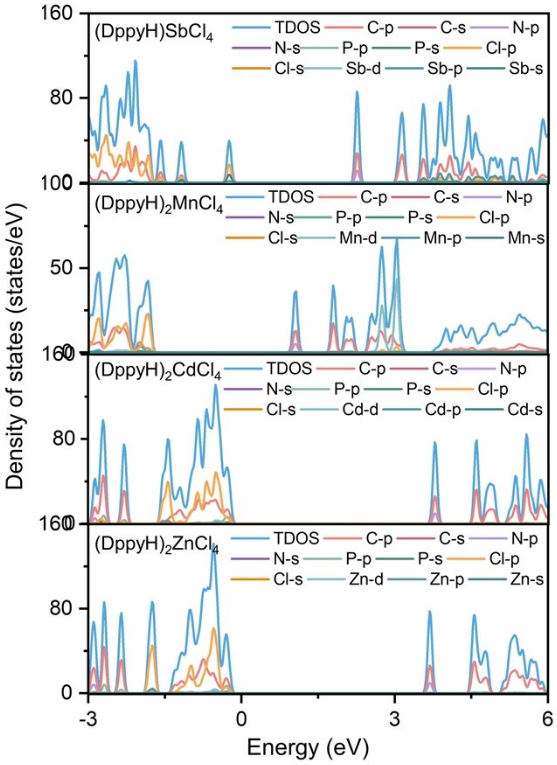

Fig. S20. The total and partial density of states of $(DppyH)_nMCl_4$ ($M = Zn^{2+}$, Cd^{2+}, Mn^{2+}, Sb^{3+}).

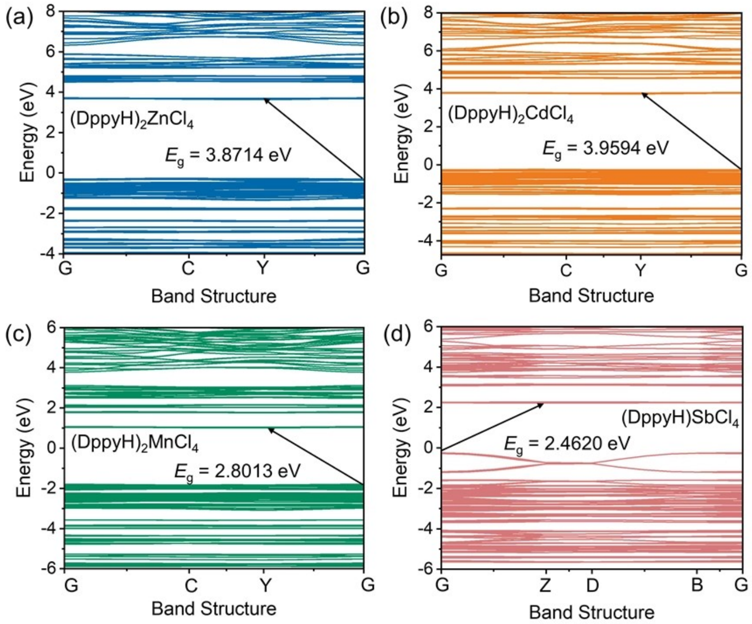

Fig. S21. (a-d) Band structures of $(DppyH)_nMCl_4$ ($M = Zn^{2+}$, Cd^{2+}, Mn^{2+}, Sb^{3+}).

References

[1] Deng, C.; Hao, S.; Liu, K.; Molokeev, M. S.; Wolverton, C.; Fan, L.; Zhou, G.; Chen, D.; Zhao, J.; Liu, Q. *J. Mater. Chem. C* 2021, *9*, 15942–15948.

[2] Chen, D.; Dai, F.; Hao, S.; Zhou, G.; Liu, Q.; Wolverton, C.; Zhao, J.; Xia, Z. *J. Mater. Chem. C* 2020, *8*, 7322-7329.

[3] Ding, X.; Zhu, G.; Geng, W.; Mikami, M.; Wang, Y. *J. Mater. Chem. C* 2015, *3*, 6676–6685.

[4] Huang, C.-H.; Wu, P.-J.; Lee, J.-F.; Chen, T.-M. *J. Mater. Chem.* 2011, *21*, 10489.

[5] Xie, R.-J.; Hirosaki, N.; Kimura, N.; Sakuma, K.; Mitomo, M. *Appl. Phys. Lett.* 2007, *90*, 191101.
